# Specialists, generalists and the shape of the ecological niche in fungi

**DOI:** 10.1111/nph.18005

**Published:** 2022-02-18

**Authors:** Daniel P. Bebber, Thomas M. Chaloner

**Affiliations:** ^1^ 8234 Department of Biosciences University of Exeter Exeter EX4 4QD UK; ^2^ 8234 CABI Wallingford OX10 8DE UK

**Keywords:** arbuscular mycorrhizal fungi, fungi, niche breadth, niche width, plant pathogens, saprotrophs

The terms ‘generalist’ and ‘specialist’ are commonly used to describe a species' ecology but their precise meaning is rarely considered. In particular, are specialists specialised in all aspects of their lives, or in only some or only one? In technical terms, is niche breadth correlated among niche dimensions? Recent research on three ecological guilds of fungi – arbuscular mycorrhizas, plant pathogens and saprotrophs – allows us to investigate the degree of co‐specialisation among niche dimensions. These studies suggest that fungi can specialise independently on climatic, edaphic and biotic conditions and resources, although evidence from saprotrophs suggests that specialisation on different climatic conditions is correlated. Quantifying niche shape has important implications for understanding how species will respond to global change.

The idea of the niche has been among the most important, yet controversial, in ecology for over a century (Chase & Leibold, 2003). The theory of evolution by natural selection considers a species' fitness in a particular environment, but it was Grinnell's (1917) paper on ‘The niche‐relationships of the California thrasher’ that explicitly defined the ecological niche as a set of environmental conditions occupied by a species. These conditions included dietary requirements, climatic tolerances and interactions with other species, such as predators. Some years later, Elton (1927) defined the niche from the perspective of a species' role in food webs and its impact on consumable resources such as prey populations. However, it was Hutchinson (1957) who introduced the revolutionary concept of the *n*‐dimensional hypervolume to define the niche and to allow, at least in theory, the niche to be empirically quantified. In Hutchinson's conceptual model, any number of limiting factors to species population growth, be they conditions such as temperature or resources such as prey, form dimensions of the hyperspace within which the species' fundamental niche sits (Blonder *et al*., 2014). The fundamental niche is defined as the niche in the absence of biotic interactions such as competition or facilitation (Carscadden *et al*., 2020). Ecological theories such as the Volterra–Gause competitive exclusion principle are conceptualised in Hutchinson's model, by stating that where the fundamental niche volumes of two species overlap, only the more competitive species will survive. The realised niche of a species is then any element of the fundamental niche that does not overlap with another, plus any parts of the intersection in which that species is the superior competitor (Hutchinson, 1957). Realised niches should therefore always be smaller than fundamental niches (Soberón & Arroyo‐Peña, 2017), except when positive interactions such as mutualism are considered (Carscadden *et al*., 2020). The concept of ecological specialisation, and comparison between specialists and generalists, can be visualised as variation in the extent of the niche along different axes or the volume of the *n*‐dimensional niche (Fig. 1). The magnitude of the niche on a particular axis is often termed the niche width or breadth (Sexton *et al*., 2017).

**Fig. 1:**
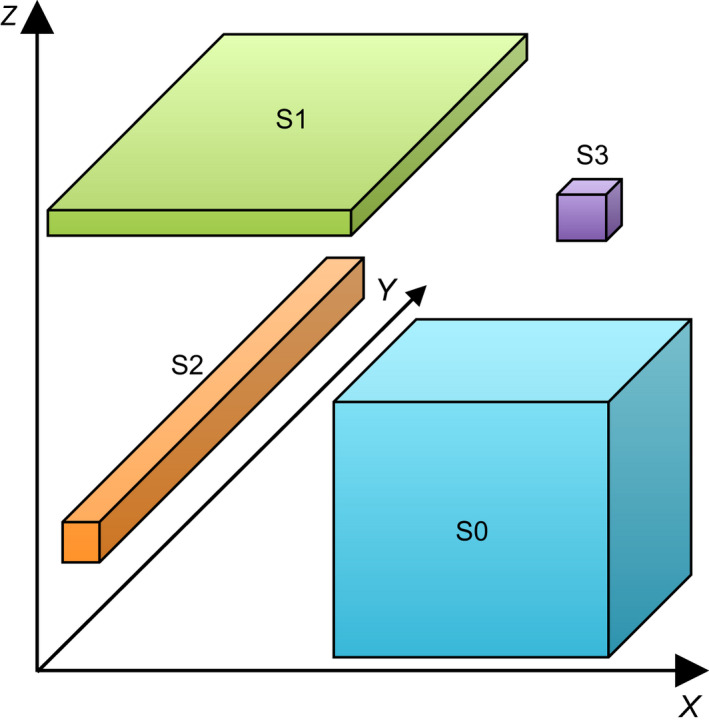
Ecological niches can be visualised as volumes in *n*‐dimensional niche space. This hypothetical example shows the niches of four species on three niche axes (*x*, *y* and *z*). Labels indicate the number of axes on which a species is specialised (i.e. has narrow niche breadth). S0 is a generalist on all axes, S3 is specialised on all axes. S1 and S2 specialise on one or two axes. Recent research on fungi allows us to consider whether specialisation on different niche axes is correlated (S0, S3) or uncorrelated (S1, S2). In some illustrations (e.g. Chaloner *et al*., 2020) the niche is depicted as a spheroid rather than a cuboid, implying interactions among niche axes.

A fundamental question in evolutionary ecology is: what determines niche breadth and how does it evolve (Sexton *et al*., 2017; Carscadden *et al*., 2020)? In the absence of competition, we might expect a species to evolve the capacity to survive under all conditions and exploit all resources. However, there must be competitive advantages to specialising, notwithstanding any biological constraints such as the implausibility of the capacity to consume prey of any size. Many models of niche evolution expect a trade‐off between performance and breadth, summed up by the phrase ‘Jack of all trades, master of none’. However, such trade‐offs remain only weakly supported by empirical evidence, and trade‐off mechanisms such as antagonistic pleiotropy operate mainly under specific conditions (Bono *et al*., 2017). Other niche evolution models indicate that specialisation can evolve without the need for trade‐offs, for example when specialists spend more time in particular environments and so beneficial alleles are fixed more rapidly than in generalist subpopulations (Whitlock, 1996).

Whereas niche breadth evolution over one niche axis has been investigated by numerous models and empirical studies (reviewed in Sexton *et al*., 2017), the relationship between niche breadths on different axes remains largely unexplored (Carscadden *et al*., 2020). Is specialisation on niche axes correlated, or uncorrelated? Is a specialist a specialist in all niche dimensions? Grinnell (1917) describes the California thrasher as preferring warm, moist environments (i.e. narrow abiotic niche axes) but having a very diverse omnivorous diet (i.e. wide resource niche axis). No correlations among niche axes were detected within the genus *Lasthenia*, an annual plant of ephemeral pools in California (Emery *et al*., 2012), whereas in global analyses of amphibians and reptiles, temperature and precipitation niche breadths were positively correlated (Bonetti & Wiens, 2014; Lin & Wiens, 2017; Liu *et al*., 2020). Whether niche breadths are correlated or not has important implications for issues such as climate change impacts on populations. For species with narrow thermal tolerances, migration to track optimal climates will be facilitated if other niche axes are broad (Carscadden *et al*., 2020).

Recent research on different guilds of fungi (Table 1) allows the question of niche breadth correlations to be addressed (Maynard *et al*., 2019; Chaloner *et al*., 2020; Davison *et al*., 2021). Writing in the *New Phytologist*, Davidson *et al* used 327 soil samples from around the world to estimate mean and standard deviation (SD) of some soil chemical properties and two climatic parameters, mean annual temperature and mean annual precipitation, for each of 230 arbuscular mycorrhizal fungal (AMF) taxa (Davison *et al*., 2021). They defined niche breath as the standard deviation of the values across sampled sites of each parameter per taxon. Strictly, geographical distributions can give an incomplete estimate of the realised niche because dispersal limitation can prevent a species from filling all of its potential range (Soberón & Nakamura, 2009; Soberón & Peterson, 2020). However, AMF are geographically widespread with little dispersal limitation (Davison *et al*., 2015; Kivlin, 2020), so this bias may be small. Davison *et al* tested for significant specialisation or generalisation by bootstrap resampling of their soil and climate data. They found that approximately half of the taxa exhibited significantly narrower niches than expected from random resampling, particularly for temperature and pH. They did not estimate correlations in niche breadths among axes, which we now present (Fig. 1a,b). We found a range of correlations among niche axes (Fig. 1a, upper triangle), from moderately negative (e.g. phosphorus and mean annual precipitation) to strongly positive (e.g. organic carbon and nitrogen). However, these correlations are themselves strongly correlated to matching correlations among the soil and climatic variables (Fig. 1b, lower triangle; Fig. 2). In other words, when abiotic conditions are correlated among sites, we see stronger correlations among niche breadths inferred from the species presences at those sites. For negatively correlated soil variables, niche breadths were ranged between −0.3 and 0.3, while for positively correlated soil variables, niche breadth correlations were generally positive but weaker than the soil correlations. The results suggest that apparent positive or negative correlations among niche axis widths could well be due to correlations among climatic and edaphic variables in this observational dataset, and that specialisation is largely independent among abiotic niche axes.

**Table 1:** Summary of niche breadth comparisons for three fungal guilds.

Guild	Plant pathogens		Arbuscular mycorrhizal fungi		Saprotrophs	
Source	Chaloner *et al*. (2020)		Davison *et al*. (2021)		Maynard *et al*. (2019)	
Taxa	209^a^		268		23	
Scale	Global^b^		Global		Regional	
Axis	Temperature	Host range	Climate^c^	Soil chemistry^d^	Temperature	Moisture
Method	Experiment	Observation	Observation	Observation	Experiment	Experiment
Niche	Fundamental	Realised	Realised	Realised	Fundamental	Fundamental

^a^ Temperature responses for growth in culture, 187 fungi and 27 oomycetes.

^b^ Probable sampling bias toward temperate climate species (Chaloner *et al*., 2021).

^c^ Mean annual temperature and mean annual precipitation.

^d^ Includes pH, organic C, N, P, K, Mg, Ca.

**Fig. 2:**
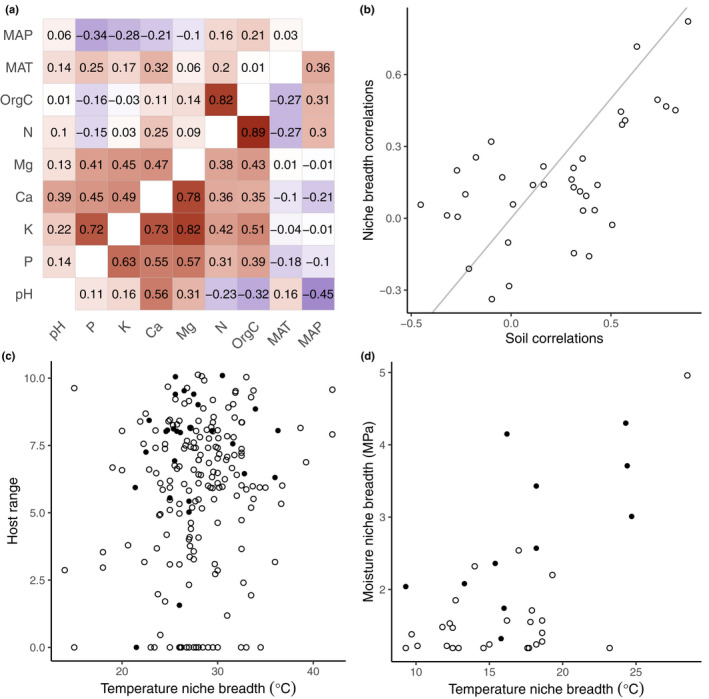
Niche breadth correlations. (a) Heat map with niche breadth correlations in upper triangle and soil property correlations in lower triangle for arbuscular mycorrhizal fungi (AMF) (data from Davison *et al*., 2021). For example, soil organic carbon (OrgC) niche breadth is strongly correlated with soil nitrogen (N) niche breadth (*r* = 0.82, upper triangle). However, soil OrgC is also strongly correlated with soil N content (*r* = 0.89, lower triangle). Red indicates a positive correlation, blue a negative correlation. MAP, mean annual precipitation; MAT, mean annual temperature. (b) Niche breadth correlations vs soil property correlations for all pairs of variables in (a). Pearson correlation 0.59 (bootstrap percentile 95% confidence interval 0.35–0.77). Line of equality in grey. (c) Host range vs temperature niche breadth (growth in culture) for fungal (open circles) and oomycete (closed circles) pathogens of plants (data from Chaloner *et al*., 2020). Host range is the log‐transformed phylogenetic diversity of known host plants. Pearson correlation 0.14 (bootstrap 95% CI 0.00–0.26). (d) Moisture niche breadth vs temperature niche breadth for AMF (data from Maynard *et al*., 2019). Pearson correlation 0.60 (bootstrap 95% CI 0.26–0.80).

Chaloner *et al*. (2020) compiled a database of niche breadths along two axes, one an abiotic condition (temperature), the other an abiotic resource (host range), for a large number of fungal and oomycete plant pathogens. They defined abiotic niche breadth as the temperature range between the minimum and maximum temperatures of some biological processes, including growth in culture and spore germination, and biotic niche breadth as the phylogenetic distances among all known plant hosts of each pathogen. They found no statistically significant positive or negative correlations among these two measures of niche breadth (once significance levels had been corrected for multiple comparisons), nor even any hints of correlations that we may not have confirmed for lack of statistical power (Fig. 1c). Acknowledging certain weaknesses in the pathogen dataset, which was compiled from older, mostly experimental results and observed rather than complete host ranges, the data indicated that specialisation on these niche axes is uncorrelated. In other words, rather than a generalist being ‘Jack of all trades, master of none’, a species can be ‘Jack of some trades, master of others’. Plant pathogens may therefore be host generalists, able to infect a diverse range of plants while also being climate specialists with narrow temperature performance curves or any other combination of niche shape.

A study on North American basidiomycetes allows us to investigate niche breadth correlations in a third guild of fungi, the saprotrophs (Maynard *et al*., 2019). Here, temperature and moisture performance curves of 37 fungal isolates in 16 genera and 23 species were experimentally determined, with temperature and moisture niche breadths estimated from those curves. The correlation between temperature and moisture niche breadths is strongly positive when all isolates are included (Fig. 1d; fig. 2 in Maynard *et al*., 2019). *Armillaria* is represented by four species, and *Armillaria gallica* by eight isolates. When *A*. *gallica* is considered alone, niche breadths remained strongly correlated (*r* = 0.67). If *Armillaria* is removed from the dataset, the correlation remains strongly positive (*r* = 0.58), largely because *Xylobolus subpileatus* is an outlier with very wide niche axes (*r* = 0.14 if *X*. *subpileatus* removed). This analysis of wood decay fungi suggests significant co‐specialisation on climate niche axes, even within species.

These three studies in different fungal (and oomycete) ecological guilds allow us to consider co‐specialisation on different niche axes and therefore the shape of the ecological niche. The AMF and saprotroph studies only considered abiotic niche axes (soil chemistry and climate), whereas the plant pathogen analysis used one biotic axis (host range) and one abiotic condition (temperature). For plant pathogens and saprotrophs, temperature and moisture niches for growth in culture were quantified experimentally and can be considered estimates of the fundamental niche. Niche breadths of the AMF were derived from geographical distributions and therefore estimate the realised niche. Given the correlations among soil properties and climate variables used to estimate niche breadth, specialisation on these factors is probably independent in AMF. For plant pathogens, the known host range estimates a subset of the fundamental niche restricted by dispersal limitation, that is plant pathogens have not reached all potential hosts (Bebber *et al*., 2014). In this fungal guild, there is no indication of co‐specialisation on climatic and biotic niche axes. In saprotrophs, there is evidence for co‐specialisation on two climatic niche axes. Therefore, taken together, we can tentatively conclude that co‐specialisation may be more likely to occur within a particular class of niche axis (e.g. climatic) than across classes (e.g. climatic and biotic).

The quantity and quality of data varied considerably among the three studies. Arbuscular mycorrhizal fungal and plant pathogens are represented by hundreds of species, but the saprotrophs by only a few. However, niche data on the saprotrophs were obtained by controlled experiment and so may be more reliable than data on the other guilds, which were obtained either by inference from geographic occurrence, or by collation of observations and historical experimental data. Further experimental studies, as well as inference from distributions, will be required to better understand niche breadth correlations (Carscadden *et al*., 2020). Temperature niche breadths are the most comparable, indicating similar variability in pathogens and saprotrophs (Fig. 1c,d). Arbuscular mycorrhizal fungal temperature niche breadths are reported as the SD of mean annual temperature at the sampled sites of each taxon (Davison *et al*., 2021). Most values lie between 1 and 10°C, and so are broadly comparable with the ranges estimated for growth in culture in the other guilds, if we assume that the majority of values lies within 2 SD of the mean. Host ranges, or biotic niche breadths, were available for pathogens but not for AMF or saprotrophs. Arbuscular mycorrhizal fungal are thought to have very low host specificity, although host preferences have been reported (van der Heijden *et al*., 2015). Host ranges of saprotrophs appear to be evolutionarily labile (Krah *et al*., 2018). White rot fungi switch frequently between generalism and angiosperm specialism, while most brown rot fungi are generalists but often switch to and from gymnosperm specialism. Phylogenetic diversities of known host plants could, in principle, be calculated for AMF using information in the Maarj*AM* database (Öpik *et al*., 2010) and for saprotrophs using the USDA Fungus–Host Distribution Database (Farr & Rossman, 2022) to allow biotic and abiotic niche breadths to be compared.

The ecological literature has had little to say on the shape of the *n*‐dimensional hypervolume, or more directly the degree of specialisation on different environmental conditions and resources within a species (Blonder *et al*., 2014; Soberón & Peterson, 2020). Major reviews of the niche concept commonly illustrate the niche as a sphere or cube rather than ovoid or oblong, implying an assumption of correlated specialisation (e.g. Chase & Leibold, 2003). Carscadden *et al*. (2020) proposed that the degree of niche breadth correlation can be determined by environmental drivers or functional constraints. Correlations in the variability of environmental conditions, rather than their levels, should determine niche breadth correlations. For example, due to opposing latitudinal gradients in plant species richness (which can be thought of as biotic variability) and annual temperature variation, we might expect temperature niche breadth to be negatively correlated with host niche breadth for plant‐associated species. Similarly, tropical plants and animals tend to have narrower thermal and wider precipitation niche breadths than temperate species (Liu *et al*., 2020). Functional constraints could inhibit the degree to which species can evolve niches to best fit environmental conditions and resources. Positive correlations in niche breadths would occur if sets of genes convey tolerances to a range of stressors, for example. This is largely speculation in the absence of data and we have little theoretical framework to consider, although new global datasets on fungal distributions derived from sequencing data may help us to answer this question (Větrovský *et al*., 2020).

Despite the apparent conceptual clarity that Hutchinson brought to the niche concept, the utility of the niche in understanding species ecology faced criticism during the 1970s and 1980s, leading ultimately to proposals that patterns of diversity and population dynamics in nature could be explained without recourse to any differences among species (Chase & Leibold, 2003). However, the close relationship between niche space and geographic distributions (Soberón & Nakamura, 2009) means that predicting the impact of global change on the biosphere requires an understanding of species' resource requirements and environmental tolerances, that is the shape of the ecological niche.

## Data Availability

Data used in the publication are openly available from the publications cited as sources in the manuscript.
